# Fractal analysis of left ventricular trabeculae in heart failure with preserved ejection fraction patients with multivessel coronary artery disease

**DOI:** 10.1186/s13244-024-01730-8

**Published:** 2024-06-18

**Authors:** Zi-Yi Gu, Bing-Hua Chen, Lei Zhao, Dong-Aolei An, Chong-Wen Wu, Song Xue, Wei-Bo Chen, Shan Huang, Yong-Yi Wang, Lian-Ming Wu

**Affiliations:** 1 Department of Cardiovascular Surgery, Renji Hospital, School of Medicine, Shanghai Jiao Tong University, Shanghai, 200127 China; 2 Department of Radiology, Renji Hospital, School of Medicine, Shanghai Jiao Tong University, Shanghai, 200127 China; 3 Philips Healthcare, Shanghai, 201103 China

**Keywords:** Heart failure, Left ventricular function, Magnetic resonance imaging, Fractal analysis

## Abstract

**Objectives:**

Endocardial trabeculae undergo varicose changes and hyperplasia in response to hemodynamic influences and are a variable phenotype reflecting changes in disease. Fractal analysis has been used to analyze the complexity of endocardial trabeculae in a variety of cardiomyopathies. The aim of this paper was to quantify the myocardial trabecular complexity through fractal analysis and to investigate its predictive value for the diagnosis of heart failure with preserved ejection fraction (HFpEF) in patients with multivessel coronary artery disease (CAD).

**Methods:**

The retrospective study population consisted of 97 patients with multivessel CAD, 39 of them were diagnosed with HFpEF, while 46 healthy volunteers were recruited as controls. Fractal dimension (FD) was obtained through fractal analysis of endocardial trabeculae on LV short-axis cine images. Logistic regression analyses were used to confirm the predictors and compare different prediction models.

**Results:**

Mean basal FD was significantly higher in patients with HFpEF than in patients without HFpEF or in the healthy group (median: 1.289; IQR: 0.078; *p* < 0.05). Mean basal FD was also a significant independent predictor in univariate and multivariate logistic regression (OR: 1.107 and 1.043, *p* < 0.05). Furthermore, adding FD to the prediction model improved the calibration and accuracy of the model (c-index: 0.806).

**Conclusion:**

The left ventricular FD obtained with fractal analysis can reflect the complexity of myocardial trabeculae and has an independent predictive value for the diagnosis of HFpEF in patients with multivessel CAD. Including FD into the diagnostic model can help improve the diagnosis.

**Critical relevance statement:**

Differences show in the complexity of endocardial trabeculae in multivessel coronary artery disease patients, and obtaining fractal dimensions (FD) by fractal analysis can help identify heart failure with preserved ejection fraction (HFpEF) patients.

**Key Points:**

- The complexity of myocardial trabeculae differs among patients with multivessel coronary artery disease.
- Left ventricular fractal dimensions can reflect the complexity of the myocardial trabecular.
- Fractal dimensions have predictive value for the diagnosis of heart failure with preserved ejection fraction.

**Graphical Abstract:**

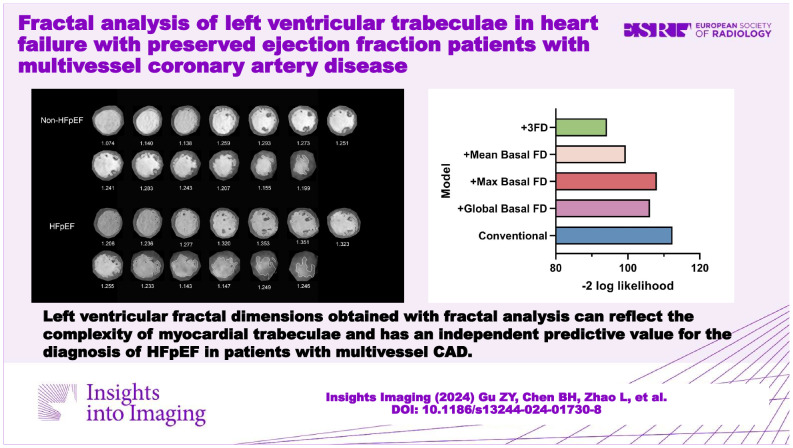

## Introduction

Heart failure (HF) is one of the most common and deadly end stages of many cardiovascular diseases [1]. Currently, heart failure affects the lives of about 40 million people worldwide [2]. Heart failure with preserved ejection fraction (HFpEF) is a form of HF whose incidence is steadily increasing every year [3, 4]. HFpEF is characterized by elevated left ventricular (LV) filling pressure due to diastolic dysfunction. Despite normal ejection fraction, patients have mild systolic dysfunction and significant limitation of systolic reserve capacity [5]. The diagnosis is challenging as it requires an evaluation of clinical history, physical examination, natriuretic peptide testing, echocardiographic data, and invasive catheterization testing to demonstrate poor cardiac output [6]. Ischemic coronary artery disease is one of the risk factors of heart failure (HF). The poor prognosis of HFpEF is thought to be related to multivessel coronary artery disease [5], but more comprehensive studies remain to be done [7–9].

Endocardial trabeculae are a complex myocardial network extending into two ventricles. In mammalian hearts, myocardial trabeculae appear in the embryonic stage. Despite the early role in the optimization of effective nutrition and gas exchange before the development of coronary artery [10], the physiological role of endocardial trabeculae in adults remains uncertain. Abnormal and excessive trabecular formation has been observed in many myocardial diseases (e.g., left ventricular noncompaction [11, 12], hypertrophic cardiomyopathy [13, 14], and pulmonary hypertension [15]). Simulation data have shown that trabeculae affect hemodynamics and improve mechanical efficiency [16, 17]. The varicose morphology of the left ventricular trabecular network is related to hemodynamic factors. It is a variable phenotype and is associated with cardiac load [18]. Currently, the changes of endocardial trabecular have not been applied to the diagnosis of clinical disease.

Fractal analysis is a sensitive, automated, and highly reproducible method for detecting subtle changes in endocardial trabeculae [12]. With cardiovascular magnetic resonance (CMR) short-axis cine sequences, the fractal dimension representing the complexity of the trabeculae can be calculated to determine their morphological changes. Several studies have applied the measurement of trabecular fractal dimension to investigate cardiac diseases [14, 19–21]. The aim of this study was to understand the complex changes in endocardial trabeculae with fractal analysis in the HFpEF patients with multivessel coronary artery disease. And to assess their predictive value as novel imaging characteristics for disease diagnosis.

## Methods

### Study population

**Fig. 1:**
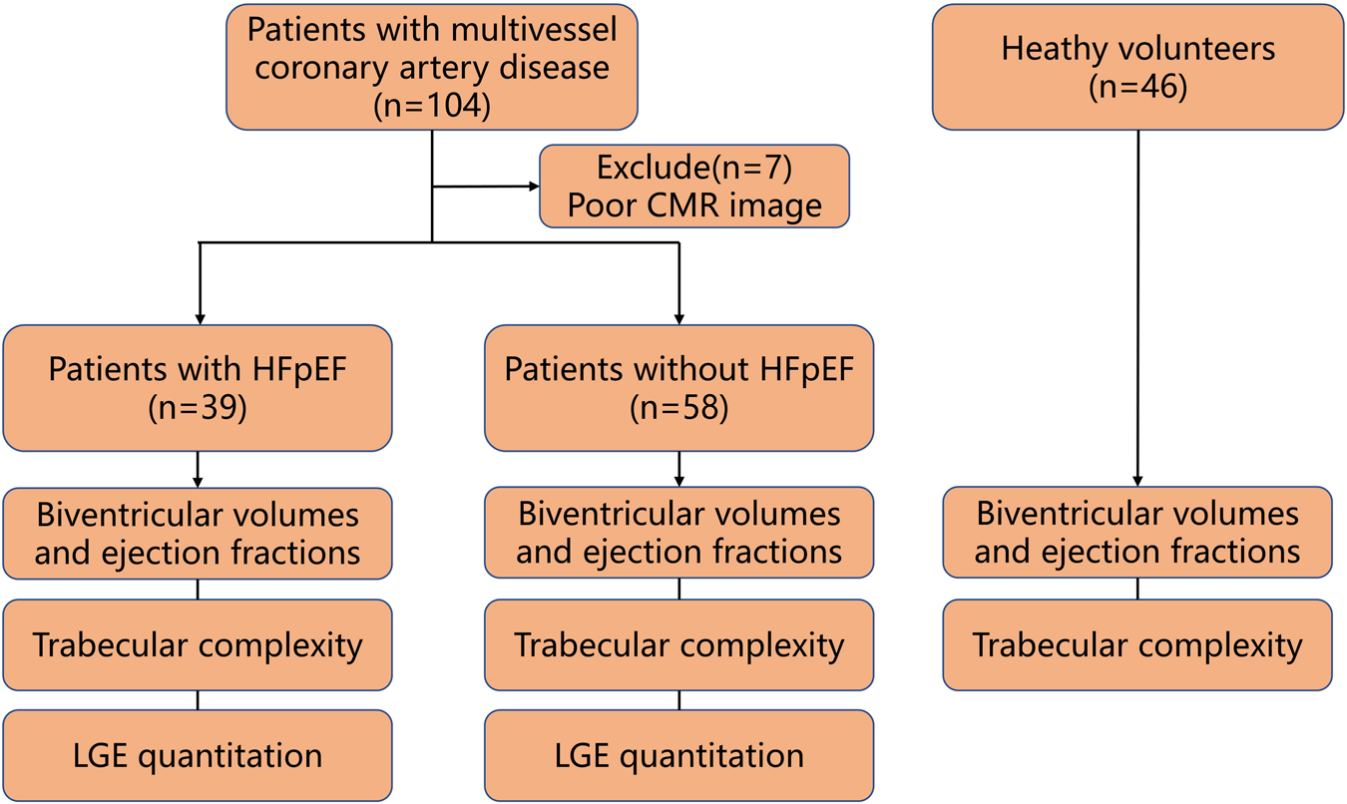
Flowchart shows numbers of patients and healthy volunteers recruited in the study

This retrospective study was approved by the local ethics committee, and all patients provided written informed consent. The study population consisted of 39 patients with multivessel coronary artery disease who were diagnosed with HFpEF, 58 patients with multivessel coronary artery disease without HFpEF, and 46 healthy volunteers as controls (Fig. 1). CAD patients were designated as having angiographic luminal narrowing > 50% in the proximal or mid part of a major coronary artery. The diagnostic criteria of HFpEF were according to the guidelines published by the European Society of Cardiology in 2016 [22]: (a) The presence of symptoms and/or signs of HF; (b) A ‘preserved’ EF (defined as LVEF ≥ 50%); (c) Elevated levels of NPs (BNP > 35 pg/mL and/or NT-proBNP > 125 pg/mL); (d) Objective evidence of other cardiac functional and structural alterations underlying HF; (e) In case of uncertainty, a stress test or invasively measured elevated LV filling pressure may be needed to confirm the diagnosis. The exclusion criteria were as follows: (a) failure to complete cardiac magnetic resonance; (b) poor image quality. Other exclusion criteria included hypertrophic cardiomyopathy, amyloidosis, congenital heart disease, advanced renal failure, or contraindication to CMR or gadolinium-based contrast agents. The healthy group were patients who were admitted to the hospital with chest pain and received a CMR examination, but no myocardial ischemia or other cardiac disease was detected after the examination.

### Cardiovascular magnetic resonance study

CMR was performed using a 3 T MRI machine (Ingenia, Philips Healthcare) using a dS torso coil anterior to the chest. For all subjects, cine images were acquired for LV function evaluation; phase-sensitive inversion recovery (PSIR) sequences were acquired for late gadolinium enhancement (LGE) assessment.

Cine imaging was performed using balanced steady-state free precession (b-SSFP) with a short-axis stack covering the whole LV and long-axis images (three, four, and two-chamber views). The imaging parameters were as follows: 2.8 ms repetition time (TR), 1.4 ms echo time (TE), 7 mm section thickness, 3 mm section gap, 300 × 300 mm field of view (FOV), 1.2 × 1.2 mm acquired matrix. LGE images were acquired with a two-dimensional (2D) PSIR sequence 10–15 min after a bolus injection of contrast medium. The injection plan was 0.15 mmol/kg of gadolinium-DTPA (Magnevist Bayer Healthcare, Berlin, Germany) with 15 mL saline flush. Imaging parameters were as follows: TR = 6.1 ms, TE = 3 ms, FOV = 300 × 300 mm, acquired matrix = 1.6 × 1.9 mm, section thickness = 7 mm, section gap = 3 mm.

### Nonfractal image analysis

All functional analysis and LGE quantitation were quantified by a commercial software CVI42 (Circle Cardiovascular Imaging, Inc.). The endocardium and epicardium of the left ventricle were semi-automatically delineated on the short axis of the ventricle at end-diastole and end-systole, and the parameters of the cardiac function were obtained after making appropriate adjustments. LGE was defined automatically by a myocardial signal intensity of a full-width at half-maximum (FWHM) method.

### Fractal analysis

**Fig. 2:**
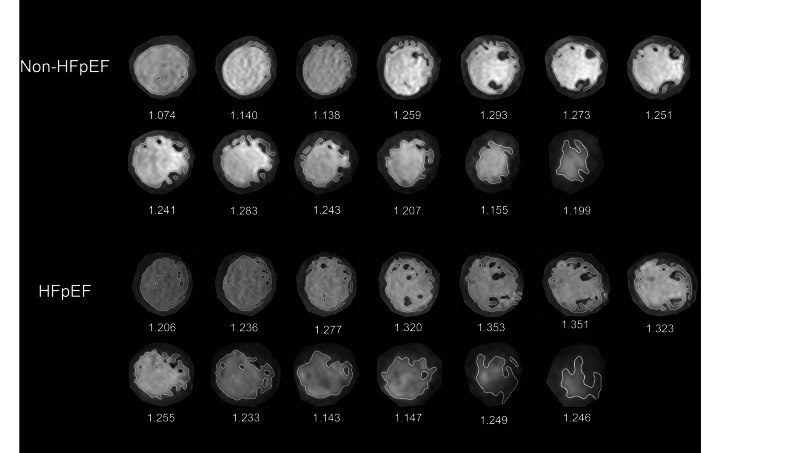
Demonstration of left ventricular fractal dimension (FD) in participants with multivessel coronary artery disease. FD extraction steps: the endocardial border was extracted at end-diastole, and endocardial trabeculae FD was subsequently calculated using a box-counting approach. Global FD was defined as an average of all FD in all measured slices. Maximal Basal FD and Maximal Apical FD were defined as the maximal value of the basal and apical slices of the ventricle. Mean Basal FD and Mean Apical FD were defined as the average values of the corresponding slices

Fractal analysis was performed by a custom-written code (FracAnalyse) in MATLAB (Math Works Inc.), which has been validated in several studies [13–15, 19, 23–25]. For each slice, the analysis procedure includes three steps: First, a region of interest was selected at the LV endocardial border on short-axis cine stacks at end-diastole. Then, the endocardial border was extracted using an image segmentation algorithm. Third, the fractal dimension (FD) value was calculated using a box-counting approach (Fig. 2). A grid of known box size was laid over the target image, and the number of boxes containing nonzero image pixels was recorded (pixels with borders = 1, background pixels = 0). This process was then repeated for multiple grids with increasing scale. As the scale increases, the number of boxes containing the object decreases exponentially and the exponent is equivalent to the FD. To quantify the exponent, the slope ( − FD) of the number of boxes against scale was estimated using linear regression. The maximum box size was set to 45% of the diameter of the endocardial border and the minimum box size was two pixels. Global FD was defined as an average of all FD in all measured slices. Maximal Basal FD and Maximal Apical FD were defined as the maximal value of the basal and apical slices of the ventricle. Mean Basal FD and Mean Apical FD were defined as the average values of the corresponding slices.

### Statistical analysis

Statistical analyses were performed using IBM SPSS statistics software (v. 24.0, IBM SPSS Inc.). The normality of the data was assessed using the Shapiro-Wilk test, and data for continuous variables were expressed as mean ± SD if they were normally distributed or as median and interquartile range (IQR) if they were non-normally distributed. Differences between groups were analyzed with the t-test or separate variance estimation t-test. Categorical variables were expressed as frequencies and percentages. Correlations between continuous variables were assessed using Pearson’s correlation coefficient. A two-sided *p-*value < 0.05 was considered significant. Univariate logistic regression analysis was used to identify variables that were significantly associated with CMR results. Variables with *p-*values  < 0.1 in the univariate analysis were subsequently included in the multiple regression using forward selection. To test whether FD improved the prediction of the clinical diagnosis, we calculated Harrell’s C-indexes and performed a likelihood ratio test.

## Results

### Demographic and baseline clinical characteristics

A total of 104 patients with multivessel coronary artery disease were recruited. Seven patients were excluded from the study because of poor short-axis cine sequences, and 97 patients were included in this study (median age 62 years; IQR 15 years; 18 females). Of these 97 patients, 39 patients with multivessel coronary artery disease were diagnosed with HFpEF (median age 64 years; IQR 14 years; eight females). In addition, 46 healthy participants (median age 35 years; IQR 21 years; 23 females) were selected as controls. Significant differences in age, gender, body mass index (BMI), brain natriuretic peptide (BNP), and heart rate were observed in patients with multivessel coronary artery disease compared to the healthy group (all *p* < 0.05), while no differences were observed between the two groups of patients with multivessel coronary artery disease. Compared to multivessel coronary artery disease patients without HFpEF (non-HFpEF group), multivessel coronary artery disease patients with HFpEF (HFpEF group) were more susceptible to hypertension (67%) and taking beta-blockers (90%) (all *p* < 0.05). In addition, the HFpEF group had a higher rate of right coronary artery occlusion (100%, *p* < 0.05). Table 1 lists the baseline characteristics of the study population.

**Table 1:** Baseline characteristics of participants

	HFpEF (*n* = 39)	Non-HFpEF (*n* = 58)	Total (*n* = 97)	Healthy (*n* = 46)
**Demographic data**				
**Age (years)**	64 (14)^b^	61.5 (19)^b^	62 (15)^b^	35 (21)
**Females**	8 (21)^b^	10 (17)^b^	18 (19)^b^	23 (50)
**BMI (kg/m**^**2**^**)**	24.9 (3.6)^b^	24.4 (5.3)	24.8 (4.1)^b^	22.4 (5.9)
**TNI (ng/mL)**	5.2 (17.9)^b^	0.09 (18.92)^b^	0.7 (18.0)	0.02 (0.02)
**BNP (pg/mL)**	153.0 (378.3)^b^	122.0 (185.0)^b^	130.0 (272.0)^b^	44.0 (51.3)
**CRP (mg/L)**	1.6 (10.9)	1.3 (6.9)	1.3 (8.5)	3.9 (3.8)
**Heart rate**	68.0 (9.0)^b^	71.0 (15.3)	70.0 (12.0)^b^	74.0 (14.3)
**Medications**				
**ACEi or ARB**	26 (67)	31 (53)	57 (59)	
**Beta Blocker**	35 (90)^a^	42 (72)	77 (79)	
**Calclum Channel Blocker**	12 (31)	10 (17)	22 (23)	
**PCI**	28 (72)	34 (59)	62 (64)	
**Past Medical History**				
**Current smoker**	18 (46)	25 (43)	43 (44)	
**Hypertension**	26 (67)^a^	24 (41)	50 (52)	
**Diabetes**	15 (39)	22 (38)	37 (38)	
**Dyslipidemia**	12 (31)	20 (35)	32 (33)	
**Previous myocardial infarction**	24 (62)	29 (50)	53 (55)	
**Chronic kidney disease**	2 (5)	2 (3)	4 (4)	
**NYHA functional class**				
**I**	5 (13)^b^	9 (16)^b^	14 (14)	46 (100)
**II**	22 (56)	41 (71)	63 (65)	
**III**	11 (28)	8 (14)	19 (20)	
**IV**	1 (3)	0 (0)	1 (1)	
**Affected vessels**				
**LAD**	38 (97)	57 (98)	95 (98)	
**LCX**	32 (82)	48 (83)	80 (83)	
**RCA**	39 (100)^a^	49 (85)	88 (91)	
**Three-vessel disease**	32 (82)	40 (69)	72 (74)	

*BMI* Body mass index, *TNI* Troponin I, *BNP* Brain natriuretic peptide, *CRP* C-reactive protein, *ACEi* angiotensin-converting enzyme inhibitor, *ARB* Angiotensin Receptor Blocker, *PCI* Percutaneous coronary intervention, *NYHA* New York Heart Association, *LAD* Left anterior descending artery, *LCX* Left circumflex artery, *RCA* Right coronary artery

Data are presented as mean ± SD or median (IQR) or n (%)

^a^ *p* < 0.05 compared with non-HFpEF

^b^ *p* < 0.05 compared with the healthy group

All CMR parameters are shown in Table 2. There were no significant differences between the groups in SV, LVEDVi, maximal apical FD, and mean apical FD. In contrast to patients with HFpEF-CAD, patients without HFpEF-CAD had significantly lower CO (median 5.2; IQR 1.6) and SVi (median 42.0; IQR 15.0), and significantly higher LVEDVi than the healthy group (all *p* < 0.05). In comparison with the non-HFpEF-CAD patients, HFpEF-CAD patients had significant differences in LVEF (56.0 ± 3.4), LVEDV (median 150.9; IQR 58.0), LVESV (median 68.0; IQR 43.0), and LVEDVi (median 73.0; IQR 33.0) (all *p* < 0.05).

**Table 2:** Cardiovascular MRI parameters of participants

	HFpEF (*n* = 39)	Non-HFpEF (*n* = 58)	Total (*n* = 97)	Healthy (*n* = 46)
**LVEF (%)**	56.0 ± 3.4^ab^	47.3 ± 16.4^b^	50.8 ± 14.6^b^	63.6 ± 5.8
**CO (L/min)**	5.5 (1.4)	5.2 (1.6)^b^	5.4 (1.5)^b^	6.1 (2.4)
**LVEDV (mL)**	150.9 (58.0)^ab^	163.0 (81.0)^b^	154.0 (57.0)^b^	127.5 (56.0)
**LVESV (mL)**	68.0 (43.0)^ab^	81.5 (64)^b^	73.0 (52.0)^b^	44.8 (22.0)
**SV (mL)**	82.0 (22.0)	73.4 (22.0)	79.0 (22.0)	81.3 (37.0)
**LV mass (g)**	122.0 (58.0)^b^	133.0 (68.0)^b^	128.58 (57)^b^	89.4 (45.0)
**LVEDV indexed to BSA (mL/m**^**2**^**)**	73.0 (33.0)^a^	86.5 (42.0)^b^	79.0 (38.0)	72.3 (19.0)
**LVESV indexed to BSA (mL/m**^**2**^**)**	49.0 (49.0)^b^	57.5 (42.0)^b^	55.0 (43)^b^	26.5 (8.0)
**SV indexed to BSA (mL/m**^**2**^**)**	44.0 (13.0)	42.0 (15.0)^b^	43.0 (14)^b^	47.5 (15.0)
**LVCI (L/min/m**^**2**^**)**	3.0 (0.6)^b^	3.0 (1.0)^b^	3.0 (0.9)^b^	3.5 (0.9)
**LV mass indexed to BSA (g/m**^**2**^**)**	61.8 (22.0)^b^	61.0 (24.0)^b^	61.0 (22)^b^	51.6 (16.0)
**LGE (%)**	12.0 (20.0)^b^	11.1 (28)^b^	12.0 (25.0)^b^	0 (0)
**Global FD**	1.266 ± 0.048^ab^	1.244 ± 0.041	1.253 ± 0.049^b^	1.235 ± 0.046
**Maximal basal FD**	1.345 (0.084)^a^	1.328 (0.056)	1.335 (0.054)	1.347 (0.075)
**Mean basal FD**	1.289 (0.078)^ab^	1.254 (0.055)	1.267 (0.058)^b^	1.258 (0.086)
**Maximal apical FD**	1.307 ± 0.059	1.299 ± 0.065	1.302 ± 0.063	1.293 ± 0.057
**Mean apical FD**	1.218 (0.085)	1.222 (0.087)	1.221 (0.088)	1.202 (0.095)

*LVEF* LV ejection fraction, *CO* Cardiac output, *LVEDV* LV end-diastolic volume, *LVESV* LV end-systolic volume, *SV* Stroke volume, *LVEDVi* LV end-diastolic volume index, *LVESVi* LV end-systolic volume index, *LVCI* LV cardiac index, *LGE* Late gadolinium enhancement, *FD* Fractal dimension

Data are presented as mean ± SD or median (IQR) or *n* (%)

^a^ *p* < 0.05 compared with non-HFpEF

^b^ *p* < 0.05 compared with the healthy group

### FD characteristics

Compared to the healthy group, global FD (1.266 ± 0.048) and mean basal FD (median 1.289; IQR 0.078) were significantly higher in HFpEF-CAD patients (*p* < 0.05), while no difference was seen in non-HFpEF-CAD patients. Compared with non-HFpEF-CAD patients, global FD, maximal basal FD (median 1.345; IQR 0.084), and mean basal FD were significantly elevated in HFpEF-CAD patients (*p* < 0.05).

**Fig. 3:**
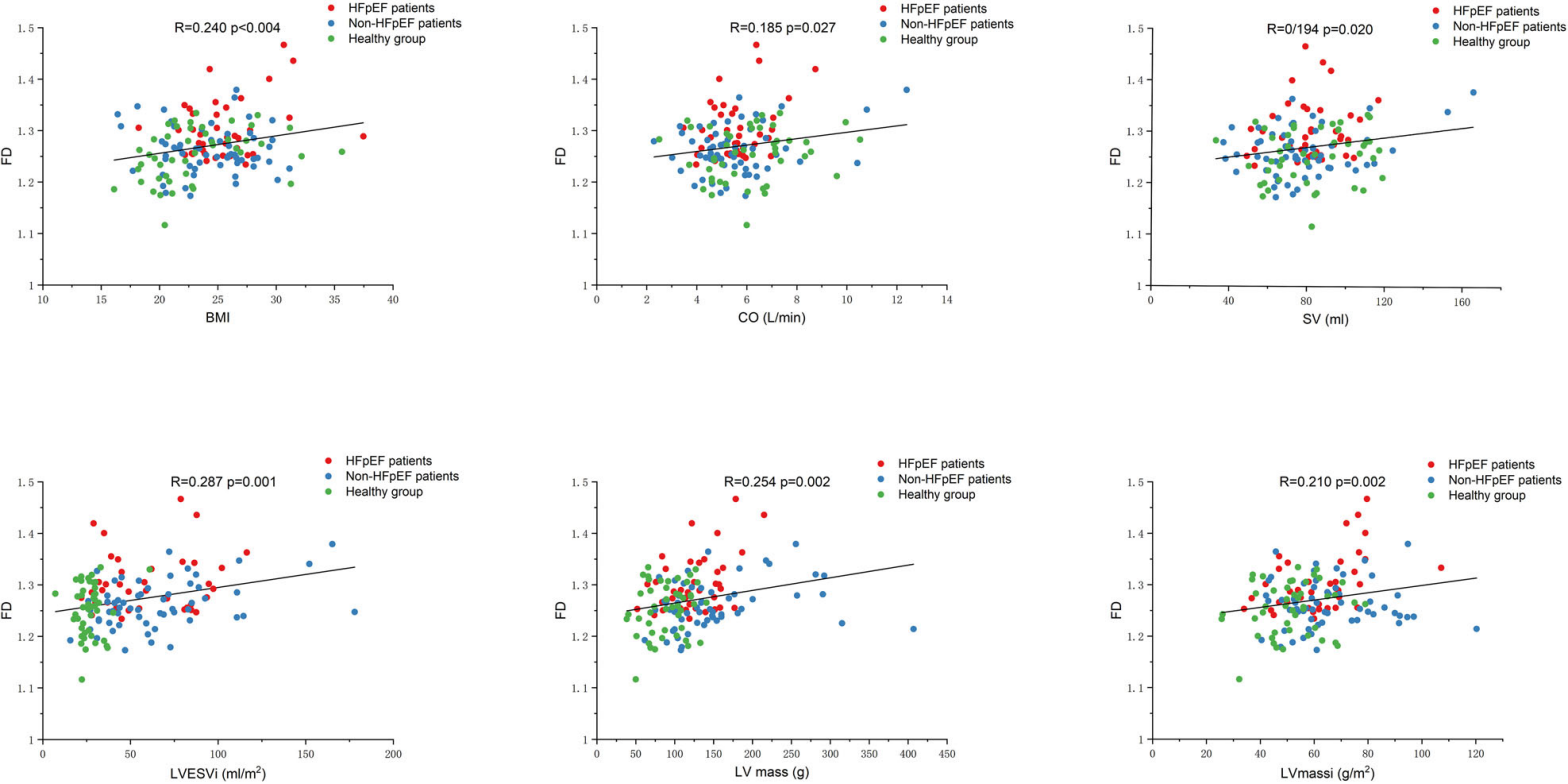
Correlations of LV mean basal FD with clinical and CMR parameters

In the correlation analysis, mean basal FD showed significant correlations with: (1) age (*r* = 0.261; *p* = 0.002); (2) BMI (*r* = 0.240; *p* = 0.004); (3) CO (*r* = 0.185; *p* = 0.027); (4) SV (*r* = 0.194; *p* = 0.020); (5) LV mass (*r* = 0.254; *p* = 0.002); (6) LVESVi (*r* = 0.287; *p* = 0.001); and (7) LV massi (*r* = 0.210; *p* = 0.012) (Supplemental Table 1 and Fig. 3). In addition, the intraobserver and inter-observer agreements showed good reproducibility of FDs measurements (Supplement Table 2).

### Logistic regression analysis

In the univariate logistic regression analysis, we included the traditional risk factors for HFpEF [26], the extent of coronary artery disease, CMR parameters, and FD as exposure factors (Table 3). The results of the analysis showed that age (OR = 1.043; *p* = 0.035), BMI (OR = 1.110; *p* = 0.095), hypertension (OR = 2.833; *p* = 0.016), LVEF (OR = 1.0048; *p* = 0.005), LVEDV (OR = 0.988; *p* = 0.011), LVESV (OR = 0.983; *p* = 0.006), LV mass (OR = 0.992; *p* = 0.060), LVEDVi (OR = 0.982; *p* = 0.011), global FD (OR = 1.012; *p* = 0.020), maximal basal FD (OR = 1.010; *p* = 0.016), and mean basal FD (OR = 1.017; *p* = 0.001) were significant univariate predictors, while maximal apical FD (OR = 1.002; *p* = 0.501) and mean apical FD (OR = 1.001; *p* = 0.740) were not.

**Table 3:** Risk factors of HFpEF in univariate and multivariate logistic analyses

	Univariate analysis		Multivariate analysis	
	OR	*p*	OR	*p*
**Age**	1.043	0.035	1.070	0.032^a^
**BMI**	1.110	0.095	1.276	0.028^a^
**BNP**	0.999	0.228		
**Hypertension**	2.833	0.016	1.905	0.293
**Diabetes**	1.023	0.958		
**Dyslipidemia**	0.844	0.703		
**Extent of coronary artery disease**				
**LAD**	0.667	0.777		
**LCX**	0.952	0.928		
**RCA**	> 10	0.999		
**Three-vessel disease**	2.057	0.153		
**LVEF**	1.048	0.005	0.937	0.487
**CO**	1.015	0.910		
**LVEDV**	0.988	0.011	1.003	0.912
**LVESV**	0.983	0.006	0.926	0.157
**SV**	1.008	0.399		
**LV mass**	0.992	0.060	0.995	0.637
**LVEDV indexed to BSA**	0.982	0.011	1.030	0.296
**LVESV indexed to BSA**	0.994	0.371		
**SV indexed to BSA**	1.014	0.450		
**LVCI**	0.990	0.968		
**LV mass indexed to BSA**	0.984	0.245		
**LGE**	0.993	0.642		
**Global FD**	1.012	0.020	1.018	0.117
**Maximal basal FD**	1.010	0.016	0.968	0.011^a^
**Mean basal FD**	1.017	0.001	1.043	0.003^a^
**Maximal apical FD**	1.002	0.501		
**Mean apical FD**	1.001	0.740		

*BMI* Body mass index, *BNP* Brain natriuretic peptide, *LAD* Left anterior descending artery, *LCX* Left circumflex artery, *RCA* Right coronary artery, *LVEF* LV ejection fraction, *CO* Cardiac output, *LVEDV* LV end-diastolic volume, *LVESV* LV end-systolic volume, *SV* Stroke volume, *LV* Left ventricular, *BSA* Body surface area, *LVCI* LV cardiac index, *LGE* Late gadolinium enhancement, *FD* Fractal dimension

^a^ *p* < 0.05 considered as statistically significant

Significant univariate parameters were added to the multivariate logistic regression analysis. Age (OR = 1.070; *p* = 0.032), BMI (OR = 1.276; *p* = 0.028), maximal basal FD (OR = 0.968; *p* = 0.011) and mean basal FD (OR = 1.043; *p* = 0.003) were identified as significant multivariate predictors.

### Performance of new prediction models

**Fig. 4:**
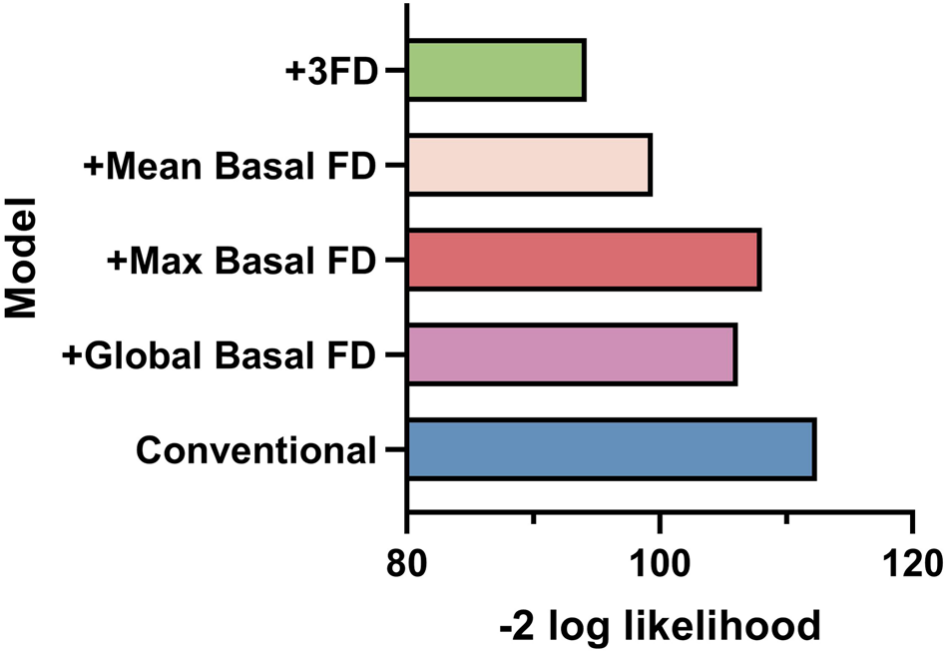
Evaluation of the accuracy and calibration of FD

The value of FD for diagnosing HFpEF in patients with multivessel coronary artery disease was assessed. FD parameters were included in the prediction model for comparison (Table 4). Compared with the conventional model with incorporating traditional risk factors such as age, BMI, BNP, hypertension, diabetes and dyslipidemia (Harrell’s C-index: 0.741; 95%CI: 0.644–0.838), incorporation of global FD (Harrell’s C-index: 0.767; 95%CI: 0.673–0.860), maximal basal FD (Harrell’s C-index: 0.753; 95%CI: 0.659–0.848), and mean basal FD (Harrell’s C-index: 0.806; 95%CI: 0.722–0.891) into the prediction model improved the Harrell’s C-index, while the simultaneous inclusion of the three FD (Harrell’s C-index: 0.824; 95%CI: 0.744-0.95) led to the highest Harrell’s C-index (Table 4). Moreover, the prediction model including FD also showed better goodness-of-fit (-2 log-likelihood ratio test; *p* < 0.05, Fig. 4). These results suggested that FD helps to improve the diagnostic model for HFpEF in patients with multivessel coronary artery disease.

**Table 4:** Harrell’s C-index for prediction

	C-index (95% CI)
**Model 1**	0.741 (0.644–0.838)
**Model 2**	0.767 (0.673–0.860)
**Model 3**	0.753 (0.659–0.848)
**Model 4**	0.806 (0.722–0.891)
**Model 5**	0.824 (0.744–0.905)

Model 1: Conventional risk factors

Model 2: Model 1 + Global FD

Model 3: Model 1 + Maximal basal FD

Model 4: Model 1 + Mean basal FD

Model 5: Model 1 + Global FD + Maximal basal FD + Mean basal FD

### Relationship between the number of vascular lesions and FD

Finally, we tried to further differentiate the HFpEF patients by the number of lesioned vessels, with 32 HFpEF patients had three lesioned vessels and 7 HFpEF patients had two lesioned vessels. The comparison of FD between HFpEF patients with different lesioned vessels did not show statistical differences, while global FD (1.266 ± 0.051) and mean basal FD (median 1.281; IQR 0.075) in patients with three lesioned vessels, and mean basal FD (median 1.305; IQR 0.058) in patients with two lesioned vessels were significantly higher than the healthy population (Supplemental Table 3). Three lesioned vessels may lead to a further increase in endocardial trabecular complexity resulting in significant changes in global FD.

## Discussion

The prevalence of HFpEF is gradually increasing. A single study showed that approximately 50% of patients with HF have HFpEF [27]. CAD is an important risk factor for HFpEF. However, there is a lack of research on HFpEF in patients with multivessel CAD. HFpEF is difficult to diagnose because of the obscure clinical presentation. Clinical diagnosis of HFpEF is mainly based on echocardiography. However, with the recent development of technology, CMR is the current gold standard imaging modality for assessing atrial and ventricular volumes, and accurately quantifying ejection fraction [28]. With its high spatial resolution, excellent signal-to-noise ratio, and inherent tomographic nature, CMR can provide morphological, functional, perfusion, viability, and tissue characteristics in a single examination. Thus, early identification of structural and functional changes in the hearts of HFpEF patients by CMR is feasible.

In this study, we used fractal analysis to assess the diagnostic value of the complexity of endocardial trabeculae in patients with multivessel coronary artery disease. First, we found that left ventricular global FD and mean basal FD were significantly higher in patients with HFpEF-CAD than in the normal population and patients with non-HFpEF-CAD. Second, LV mean basal FD was significantly correlated with age, BMI, and LV mass. Third, LV mean basal FD was a strong predictor of HFpEF in multivariate logistic correlation analysis. Fourth, the inclusion of FD in the prediction model significantly improved the diagnostic value and goodness-of-fit of the model compared with the conventional prediction model.

The incidence of HFpEF is increasing, and 4.9% of the general population over 60 years of age is diagnosed with HFpEF [29]. To date, treatment options are relatively limited, possibly due to the pathophysiological heterogeneity within the broader clinical spectrum. Therefore, effective diagnostic methods are needed to facilitate individualized treatment [6]. With substantial improvements in both spatial and temporal resolutions in cardiac imaging modalities, complex ventricular anatomy can also be visualized, making endocardial trabecular border tracing an interesting entry point [30]. Excessive proliferation of ventricular trabeculae has been found to be associated with multivessel CAD [25, 30, 31]. Given that left ventricular compensation is inevitable for the heart to maintain normal ventricular function, trabecular hyperplasia and changes in complexity will be potentially used for early diagnosis in patients with HFpEF [32]. Fractal analysis has been demonstrated as a reliable method to assess trabecular complexity in several studies. Our repeatability test of FDs measurements also proved that fractal analysis has good reproducibility. Captur et al [24] found that changes in LV trabeculae could be assessed using a semi-automatic tool, and abnormal trabeculae are often a disease feature. Wang et al [14] found that LV apical fractal dimension provided incremental prognostic value for patients with hypertrophic cardiomyopathy by fractal analysis. Dawes et al [15] found that fractal analysis can also be used in the right ventricle, where the complexity of the RV trabeculae was a marker of elevated afterload in patients with pulmonary hypertension.

In this study, LV global FD and mean basal FD showed significant differences in HFpEF patients, but the difference in global FD was not statistically significant in logistic regression analysis, possibly due to the major compensatory function occurring in the middle or basal of the left ventricle during maintenance of LV function. Wang et al [33] estimated diastolic myocardial stiffness and stress by personalized biomechanical modeling and analysis techniques. They found that heart failure patients had higher myofiber stress in mid-ventricular region. This is consistent with our finding. Camporeale et al [19] found a positive correlation between FD, age, and LVmassi, which is consistent with our finding. Mean basal FD was also positively associated with traditional HFpEF risk factors such as BMI, suggesting that there are multiple adaptive mechanisms underlying increased trabecular complexity in patients. Hwang et al [7] found similar changes in LV function and outcomes in HFpEF patients without CAD and patients with single-vessel CAD, so they speculated that the adverse effects of CAD on HFpEF may be related to multivessel disease. In the present study, mean basal FD in HFpEF patients with three or two-vessel disease showed significant differences compared with the healthy population, and patients with three-vessel disease had more significant differences, suggesting that multivessel CAD is involved in the development of HFpEF and FD is a valid assessment tool. The accuracy and goodness-of-fit of the model was improved before including FD into the diagnostic model, especially for mean basal FD. The combined inclusion of three FD yielded the best diagnostic model.

This study had some limitations. First, this was a single-center study from China with a small sample size, which also led to a low number of female patients with HFpEF in this study. Additional studies from multiple centers are needed to verify the performance of the proposed diagnostic model. Second, the study design was cross-sectional and lacked long-term measurements of FD in patients to illustrate the progressive change of trabecular complexity in HFpEF development. Third, subdividing the CAD patients into HFrEF-CAD, HFpEF-CAD, and nonHF-CAD might better reflect the trend of FD changes.

In summary, HFpEF patients with multivessel CAD have changes in myocardial trabecular complexity. The left ventricular FD obtained with fractal analysis can reflect the complexity of myocardial trabeculae and has an independent predictive value for the diagnosis of HFpEF in patients with multivessel CAD. Including FD into the diagnostic model can help improve the diagnosis.

### Supplementary information

ELECTRONIC SUPPLEMENTARY MATERIAL

## Data Availability

The data used in the study are available from the corresponding author on reasonable request.

## Abbreviations

BMI: Body mass index
BNP: Brain natriuretic peptide
BSA: Body surface area
CAD: Coronary artery disease
CMR: Cardiovascular magnetic resonance imaging
CO: Cardiac output
FD: Fractal dimension
HF: Heart failure
HFpEF: Heart failure with preserved ejection fraction
IQR: Interquartile range
LGE: Late gadolinium enhancement
LV: Left ventricular
LVEDV: LV end-diastolic volume
LVEDVi: LV end-diastolic volume index
LVEF: LV ejection fraction
LVESV: LV end-systolic volume
LVESVi: LV end-systolic volume index
PSIR: Phase-sensitive inversion recovery
SV: Stroke volume

## References

[1] McMurray JJ, Pfeffer MA (2005). Heart failure. Lancet.

[2] Baman JR, Ahmad FS (2020). Heart failure. JAMA.

[3] Zhang X, Zhou Y, Wei N (2022). A bibliometric analysis of heart failure with preserved ejection fraction from 2000 to 2021. Curr Probl Cardiol.

[4] Dunlay SM, Roger VL, Redfield MM (2017). Epidemiology of heart failure with preserved ejection fraction. Nat Rev Cardiol.

[5] Borlaug BA (2014). The pathophysiology of heart failure with preserved ejection fraction. Nat Rev Cardiol.

[6] Borlaug BA (2020). Evaluation and management of heart failure with preserved ejection fraction. Nat Rev Cardiol.

[7] Hwang SJ, Melenovsky V, Borlaug BA (2014). Implications of coronary artery disease in heart failure with preserved ejection fraction. J Am Coll Cardiol.

[8] Patel MR, Calhoon JH, Dehmer GJ (2017). ACC/AATS/AHA/ASE/ASNC/SCAI/SCCT/STS 2017 appropriate use criteria for coronary revascularization in patients with stable ischemic heart disease: a report of the American College of Cardiology Appropriate Use Criteria Task Force, American Association for Thoracic Surgery, American Heart Association, American Society of Echocardiography, American Society of Nuclear Cardiology, Society for Cardiovascular Angiography and Interventions, Society of Cardiovascular Computed Tomography, and Society of Thoracic Surgeons. J Am Coll Cardiol.

[9] Felker GM, Shaw LK, O’Connor CM (2002). A standardized definition of ischemic cardiomyopathy for use in clinical research. J Am Coll Cardiol.

[10] Sedmera D, Pexieder T, Hu N (1997). Developmental changes in the myocardial architecture of the chick. Anat Rec.

[11] Petersen SE, Selvanayagam JB, Wiesmann F (2005). Left ventricular non-compaction: insights from cardiovascular magnetic resonance imaging. J Am Coll Cardiol.

[12] Captur G, Muthurangu V, Cook C (2013). Quantification of left ventricular trabeculae using fractal analysis. J Cardiovasc Magn Reson.

[13] Captur G, Lopes LR, Patel V (2014). Abnormal cardiac formation in hypertrophic cardiomyopathy: fractal analysis of trabeculae and preclinical gene expression. Circ Cardiovasc Genet.

[14] Wang J, Li Y, Yang F (2021). Fractal analysis: prognostic value of left ventricular trabecular complexity cardiovascular MRI in participants with hypertrophic cardiomyopathy. Radiology.

[15] Dawes TJW, Cai J, Quinlan M (2018). Fractal analysis of right ventricular trabeculae in pulmonary hypertension. Radiology.

[16] Kulp S, Gao M, Zhang S (2011). Using high resolution cardiac CT data to model and visualize patient-specific interactions between trabeculae and blood flow. Med Image Comput Comput Assist Inter.

[17] Fatemifar F, Feldman MD, Clarke GD et al (2019) Computational modeling of human left ventricle to assess the effects of trabeculae carneae on the diastolic and systolic functions. J Biomech Eng 141:091014 10.1115/1.4043831 31116359

[18] Captur G, Syrris P, Obianyo C (2015). Formation and malformation of cardiac trabeculae: biological basis, clinical significance, and special yield of magnetic resonance imaging in assessment. Can J Cardiol.

[19] Camporeale A, Moroni F, Lazzeroni D (2022). Trabecular complexity as an early marker of cardiac involvement in Fabry disease. Eur Heart J Cardiovasc Imaging.

[20] Captur G, Moon JC (2021). Top cats often begin as underdogs: the ascent of trabecular fractal analysis with cardiac MRI. Radiology.

[21] Krupickova S, Hatipoglu S, DiSalvo G (2021). Left ventricular noncompaction in pediatric population: could cardiovascular magnetic resonance derived fractal analysis aid diagnosis?. J Cardiovasc Magn Reson.

[22] Ponikowski P, Voors AA, Anker SD (2016). 2016 ESC Guidelines for the diagnosis and treatment of acute and chronic heart failure: The Task Force for the diagnosis and treatment of acute and chronic heart failure of the European Society of Cardiology (ESC). Developed with the special contribution of the Heart Failure Association (HFA) of the ESC. Eur J Heart Fail.

[23] Yu S, Chen X, Yang K (2022). Correlation between left ventricular fractal dimension and impaired strain assessed by cardiac MRI feature tracking in patients with left ventricular noncompaction and normal left ventricular ejection fraction. Eur Radio.

[24] Captur G, Radenkovic D, Li C (2017). Community delivery of semiautomated fractal analysis tool in cardiac mr for trabecular phenotyping. J Magn Reson Imaging.

[25] Captur G, Zemrak F, Muthurangu V (2015). Fractal analysis of myocardial trabeculations in 2547 study participants: multi-ethnic study of atherosclerosis. Radiology.

[26] Pieske B, Tschope C, de Boer RA (2019). How to diagnose heart failure with preserved ejection fraction: the HFA-PEFF diagnostic algorithm: a consensus recommendation from the Heart Failure Association (HFA) of the European Society of Cardiology (ESC). Eur Heart J.

[27] Ceia F, Fonseca C, Mota T (2002). Prevalence of chronic heart failure in Southwestern Europe: the EPICA study. Eur J Heart Fail.

[28] Chamsi-Pasha MA, Zhan Y, Debs D (2020). CMR in the evaluation of diastolic dysfunction and phenotyping of HFpEF. Curr Role Future Perspect JACC Cardiovasc Imaging.

[29] van Riet EE, Hoes AW, Wagenaar KP (2016). Epidemiology of heart failure: the prevalence of heart failure and ventricular dysfunction in older adults over time. A systematic review. Eur J Heart Fail.

[30] Chuang ML, Gona P, Hautvast GL (2012). Correlation of trabeculae and papillary muscles with clinical and cardiac characteristics and impact on CMR measures of LV anatomy and function. JACC Cardiovasc Imaging.

[31] Patel AR, Mor-Avi V (2012). Are trabeculae and papillary muscles an integral part of cardiac anatomy: or annoying features to exclude while tracing endocardial boundaries?. JACC Cardiovasc Imaging.

[32] Brandes R, Maier LS, Bers DM (1998). Regulation of mitochondrial [NADH] by cytosolic [Ca2+] and work in trabeculae from hypertrophic and normal rat hearts. Circ Res.

[33] Wang ZJ, Wang VY, Bradley CP (2018). Left ventricular diastolic myocardial stiffness and end-diastolic myofibre stress in human heart failure using personalised biomechanical analysis. J Cardiovasc Transl Res.

