# Supplementary material for: Fractal analysis of left ventricular trabeculae in heart failure with preserved ejection fraction patients with multivessel coronary artery disease
Source: Insights Imaging. 2024 Jun 18;15:148. doi: 10.1186/s13244-024-01730-8 (PMC11183012; doi:10.1186/s13244-024-01730-8)
Supplement: Supplementary file 1 — ELECTRONIC SUPPLEMENTARY MATERIAL [file 13244_2024_1730_MOESM1_ESM.pdf]

Fractal Analysis of Left Ventricular Trabeculae in Heart Failure with Preserved Ejection Fraction Patients with Multivessel Coronary Artery Disease

ELECTRONIC SUPPLEMENTARY MATERIAL

Supplemental Table 1. Correlations of total fractal dimension with CMR and clinical parameters						
		Global FD	Maximal basal FD	Mean basal FD	Maximal apical FD	Mean apical FD
**Age, years**	r	0.106	0.190 *	0.261 *	-0.112	-0.052
	p	0.207	0.023	0.002	0.184	0.534
**BMI, kg/m2**	r	0.340 *	0.243 *	0.240 *	0.276 *	0.308 *
	p	<0.001	0.003	0.004	0.001	<0.001
**TNI (ng/ml)**	r	0.172 *	-0.002	-0.029	0.217 *	0.276 *
	p	0.040	0.978	0.735	0.009	0.001
**BNP (pg/ml)**	r	0.097	0.017	0.081	-0.024	0.081
	p	0.249	0.844	0.334	0.778	0.338
**CRP (mg/l)**	r	0.206 *	0.106	0.142	0.187 *	0.185 *
	p	0.014	0.210	0.091	0.026	0.027
**Heart rate**	r	-0.022	0.036	-0.032	-0.018	-0.016
	p	0.790	0.668	0.709	0.831	0.849
**LVEF**	r	-0.216 *	-0.090	-0.034	-0.253 *	-0.305 *
	p	0.009	0.287	0.687	0.002	<0.001
**CO**	r	0.164	0.118	0.185 *	0.074	0.087
	p	0.051	0.162	0.027	0.378	0.301
**LVEDV**	r	0.240 *	0.084	0.122	0.209 *	0.274 *
	p	0.004	0.319	0.148	0.012	0.001
**LVESV**	r	0.176 *	0.044	0.047	0.178 *	0.240 *
	p	0.035	0.602	0.581	0.033	0.004
**SV**	r	0.178 *	0.105	0.194 *	0.095	0.107
	p	0.034	0.212	0.020	0.257	0.202
**LV mass**	r	0.246 *	0.208 *	0.254 *	0.137	0.168 *
	p	0.003	0.012	0.002	0.102	0.045
**LVEDV indexed to BSA**	r	0.117	0.005	-0.005	0.137	0.189 *
	p	0.163	0.954	0.950	0.102	0.024
**LVESV indexed to BSA**	r	0.196 *	0.192 *	0.287 *	0.052	0.070
	p	0.019	0.022	0.001	0.539	0.404
**SV indexed to BSA**	r	0.033	0.029	0.100	-0.013	-0.031
	p	0.695	0.734	0.236	0.880	0.715
**LVCI**	r	0.014	0.023	0.091	-0.049	-0.060
	p	0.868	0.787	0.281	0.561	0.478
**LV mass indexed to BSA**	r	0.254 *	0.233 *	0.210 *	0.190 *	0.216 *
	p	0.002	0.005	0.012	0.023	0.010
**LGE**	r	0.124	0.009	-0.003	0.147	0.197 *
	p	0.140	0.911	0.974	0.079	0.018

*p < 0.05 considered as statistically significant.

Supplemental Table 2. Inter and intra-class correlation coefficient of FDs		
	Interclass-ICC (95% CI)	Intraclass-ICC (95% CI)
**Global FD**	0.978 (0.969, 0.985)	0.989 (0.985, 0.993)
**Maximal basal FD**	0.939 (0.912, 0.957)	0.969 (0.956, 0.979)
**Mean basal FD**	0.966 (0.952, 0.976)	0.983 (0.976, 0.988)
**Maximal apical FD**	0.937 (0.912, 0.956)	0.968 (0.954, 0.977)
**Mean apical FD**	0.966 (0.951, 0.976)	0.983 (0.975, 0.988)

Supplemental Table 3. Comparison of lesioned vessels			
	Three-vessel disease HFpEF (n=32)	Two-vessel disease HFpEF (n=7)	Healthy (n=46)
**Global FD**	1.266±0.051 †	1.269±0.027	1.235±0.046
**Maximal basal FD**	1.343(0.092)	1.349(0.068)	1.347(0.075)
**Mean basal FD**	1.281(0.075) †	1.305(0.058) †	1.258(0.086)
**Maximal apical FD**	1.305±0.065	1.320±0.025	1.293±0.057
**Mean apical FD**	1.216(0.105)	1.224(0.050)	1.202(0.095)

Data are presented as mean ± SD or median (IQR) or n (%)

*p< 0.05 compared with two-vessel disease patients

†p< 0.05 compared with the healthy group
